# Oxidative Stress, Oxidative Damage, and Cell Apoptosis: Toxicity Induced by Arecoline in *Caenorhabditis elegans* and Screening of Mitigating Agents

**DOI:** 10.3390/toxins16080352

**Published:** 2024-08-12

**Authors:** Kaiping Xiang, Bing Wang, Lanying Wang, Yunfei Zhang, Hanzeng Li, Yanping Luo

**Affiliations:** 1 School of Tropical Agriculture and Forestry, Hainan University, Haikou 570228, China; 22220951320045@hainanu.edu.cn (K.X.); 21220951320089@hainanu.edu.cn (B.W.); 990992@hainanu.edu.cn (L.W.); 994302@hainanu.edu.cn (Y.Z.); 2 School of Environmental Science and Engineering, Hainan University, Haikou 570228, China

**Keywords:** arecoline, *C. elegans*, ROS, apoptosis, screening of mitigating agents

## Abstract

As the areca nut market is expanding, there is a growing concern regarding areca nut toxicity. Areca nut alkaloids are the major risky components in betel nuts, and their toxic effects are not fully understood. Here, we investigated the parental and transgenerational toxicity of varied doses of areca nut alkaloids in *Caenorhabditis elegans*. The results showed that the minimal effective concentration of arecoline is 0.2–0.4 mM. First, arecoline exhibited transgenerational toxicity on the worms’ longevity, oviposition, and reproduction. Second, the redox homeostasis of *C. elegans* was markedly altered under exposure to 0.2–0.4 mM arecoline. The mitochondrial membrane potential was thereafter impaired, which was also associated with the induction of apoptosis. Moreover, antioxidant treatments such as lycopene could significantly ameliorate the toxic effects caused by arecoline. In conclusion, arecoline enhances the ROS levels, inducing neurotoxicity, developmental toxicity, and reproductive toxicity in *C. elegans* through dysregulated oxidative stress, cell apoptosis, and DNA damage-related gene expression. Therefore, the drug-induced production of reactive oxygen species (ROS) may be crucial for its toxic effects, which could be mitigated by antioxidants.

## 1. Introduction

The areca nut originates from Southeast Asia, and is now mainly produced and consumed in tropical areas of China. As one of the four major ingredients of the southern branch of traditional Chinese medicine, the areca nut is widely used for therapeutic and psychoactive purposes [1] and has been consumed for more than 1800 years in many countries such as China, Thailand, Malaysia, and Indonesia. In 2019, areca nut consumers exceeded 600 million [2]. The major effective constituent of areca nut is arecoline, which is ranked as the fourth most commonly used human psychoactive substance in the world [3,4]. However, arecoline has been found to have significant toxic side effects [5]. According to previous studies [6], arecoline is a potent agonist of muscarinic acetylcholine receptors and exerts various effects on the central nervous system (CNS) including stimulation, alertness, and excitation. It has also been found that arecoline triggers motor hyperactivity in zebrafish and is able to activate various muscarinic acetylcholine receptors [7]. It has also been reported that arecoline induces excitatory responses in dopaminergic neurons in the ventral tegmental area of anesthetized rats [5]. A significant reduction in hatching and survival rates, growth retardation, and impaired swimming ability were observed with embryonic exposure to areca nut alkaloids. In addition, areca nut alkaloids induced apoptotic defects in mouse oocytes and had toxic effects on mouse embryos [8,9]. It is evident that areca nut alkaloids can cause neurotoxicity, developmental toxicity, and even reproductive toxicity to organisms. Thus, the side effects of the consumption of areca nut should be further investigated.

The toxic effect associated with the medical or dietary consumption of areca nuts depends on the dosage. Additionally, the contribution of the chemical effects of arecoline and the direct oral damage due to physical friction needs to be separately analyzed. The oral concentration of arecoline from different donors varies greatly depending on chewing habits. Salivary arecoline was found to exceed the threshold of 0.1–10 μg/mL for all participants during areca nut chewing in a clinical study [10]. It was also found that the maximum fluid concentration of arecoline in the oral cavity of volunteers during areca nut could reach up to 83.6 μg/mL, much higher than the 10 μg/mL (60 µM) concentration that is capable of stimulating arecoline-dependent signaling in cultured cells [11,12]. Consistently, another study also showed that the concentration of arecoline in the saliva of areca nut chewers usually ranged from 40 μM to 400 μM [12].

*C. elegans* is a common genetics model used to dissect the pharmacological mechanism of natural products or drugs [13,14]. It has a short lifespan, fixed cell lineages, conserved signaling pathways, and is easy for versatile genetic manipulations [15]. Due to a lack of systematic studies on areca nut toxicity, we assessed the areca nut toxicity in *C. elegans*. To this end, we selected 0.04 mM, 0.2 mM, and 0.4 mM as the test concentrations based on the salivary concentrations detected in human cohorts, and evaluated the direct and transgenerational impact of arecoline on *C. elegans* neuronal activity [16], development, and reproduction. This study systematically evaluated the behavioral characteristics of *C. elegans* under arecoline salivary concentration conditions and conducted comprehensive biochemical analyses of neural, reproductive, and oxidative damage, spanning the organismal to molecular levels. Additionally, the detoxification mechanism of lycopene was assessed as a countermeasure. The findings provide theoretical support for the accurate risk assessment of arecoline and offer scientific foundations for the prevention and intervention of its toxic effects.

## 2. Results

### 2.1. Transgenerational Impact of Arecoline on the Timing of Lifespan and Development of C. elegans

**Figure 1:**
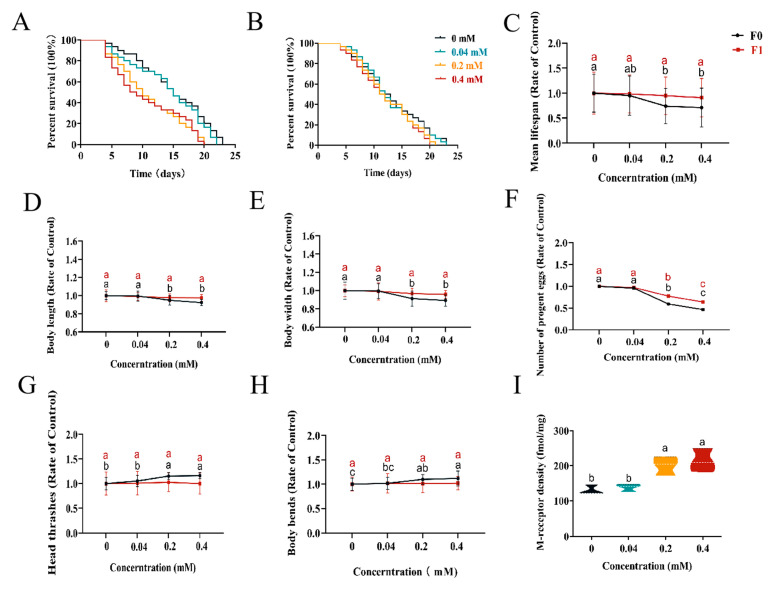
Effect of arecoline on the toxicity to *C. elegans.* (**A**) F0 generation lifespan; (**B**) F1 generation lifespan; (**C**) average longevity; (**D**) body length; (**E**) body width; (**F**) average egg production; (**G**) head thrashes; (**H**) body bends; (**I**) muscarinic acetylcholine receptor content. Data are presented as mean ± SEM. Values followed by the different lowercase letters within a row represent the significant difference (Tukey tests, *p* < 0.05). One-way ANOVA with Tukey post hoc test.

To determine the toxicity of arecoline, we set out to measure the lifespan of *C. elegans* after arecoline treatments. The maximum lifespan of F0 generation nematodes was 23 d, 22 d, 21 d, and 20 d after incubation with 0 mM, 0.04 mM, 0.2 mM, and 0.4 mM, respectively (Figure 1A); the calculated mean lifespan was 14.9 d, 14.17 d, 11.03 d, and 10.6 d, respectively (Figure 1C). In addition, the maximum lifespan of F1 generation *C. elegans* at different concentrations was 23, 23, 21, and 20 days (Figure 1B); the mean lifespan was 13.1, 12.9, 12.4, and 11.9 days, respectively. Compared with the control, the mean lifespan of *C. elegans* in the arecoline treatment was reduced by 0.73, 3.87, and 4.3 days, respectively. These results indicate that arecoline exerts a mild defect on *C. elegans* longevity, which can at least be inherited for one generation.

To understand the developmental effects caused by arecoline, the body length and width of *C. elegans* were examined after different concentrations of arecoline exposure. The results (Figure 1D,E) showed that the body length and width of the F0 generation decreased with the increase in treatment concentration at 72 h. The treatments of 0.2 mM and 0.4 mM showed the most pronounced difference from the control. However, the length and body width of the F1 generation recovered back to normal and were not significantly different from the control. Therefore, unlike longevity, the development of *C. elegans* was impaired by arecoline but did not exhibit a transgenerational effect.

### 2.2. Effect of Arecoline on Fertility of C. elegans

Arecoline may jeopardize the reproduction of *C. elegans*. To test this possibility, we recorded the brood size of *C. elegans* with or without exposure to arecoline. The results showed that the brood size of the F0 generation of *C. elegans* decreased proportionally to the increase in arecoline concentration (Figure 1F), meaning that egg production after the 0.2 mM and 0.4 mM treatments was significantly different from the control. Interestingly, the average egg production of the F1 generation was also markedly impaired when their parents were exposed to arecoline, implying a transgenerational mechanism of arecoline in deteriorating fertility.

### 2.3. Neurotoxicity of Arecoline on C. elegans

To assess the neuronal toxicity of arecoline, we measured the effects of different concentrations of arecoline exposure on the head thrashes and body bending of *C. elegans*. The results (Figure 1G,H) showed that the number of head thrashes and body bends of the F0 generation of *C. elegans* showed an increasing trend with increasing concentrations when incubated with the agents for 3 days. The treatments of 0.2 mM and 0.4 mM were significantly different from the control. In contrast, the frequency of head thrashes and body bends of the F1 generation of *C. elegans* did not change significantly with the concentration and were not significantly different from the control at the test concentrations. Therefore, the motor neuron defects resulting from arecoline treatment are not transgenerational. Besides the behavioral marker, we also measured the content of muscarinic acetylcholine receptors (Figure 1I) in *C. elegans* with the arecoline treatment. The number of muscarinic acetylcholine receptors increased proportionally to the arecoline concentration. The treatments of 0.2 mM and 0.4 mM showed the most marked increase from the control. This is consistent with the hyperexcitation behavior that is consequent to arecoline treatment.

### 2.4. Effect of Arecoline on Oxidative Stress C. elegans

**Figure 2:**
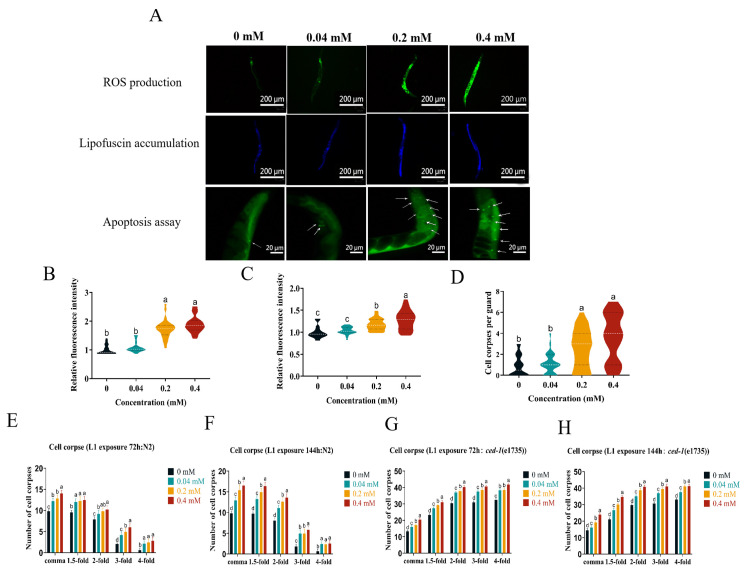
Effects of arecoline on ROS, lipofuscin accumulation, and gonad cell corpses in *C. elegans*. (**A**) Fluorescence pictures of ROS and lipofuscin accumulation and the gonad cell corpses assay. (**B**) Quantification of ROS accumulation. (**C**) Quantification of lipofuscin accumulation. (**D**) Quantification of gonad cell corpses. (**E**,**F**) Cell corpses were counted in N2. (**G**,**H**) Cell corpses were counted in the *ced-1(e1735)* strains. Values followed by the different lowercase letters within a row represent the significant difference (Tukey tests, *p <* 0.05). One-way ANOVA with Tukey post hoc test.

To further explore the mechanism behind arecoline toxicity, we analyzed the ROS levels in *C. elegans* with the help of fluorescence microscopy. The results (Figure 2A) showed that the ROS levels in *C. elegans* increased gradually with the increase in the concentration of the treatment group after arecoline exposure. The ROS content in *C. elegans* increased by 1.09-fold, 1.73-fold, and 1.89-fold under the 0.04 mM, 0.2 mM, and 0.4 mM treatments, respectively (Figure 2B), and the ROS content was significantly different from the control with the 0.2 mM and 0.4 mM treatments.

Lipofuscin, which is mainly composed of oxidized lipids and proteins, is an important sign of aging in *C. elegans*. The rise of reactive oxygen species in organisms will accelerate the oxidation process of lipids and proteins, resulting in increased lipofuscin content in the body. We measured the effects of arecoline exposure at different concentrations on lipofuscin accumulation in *C. elegans*. The results showed that the fluorescence intensity in *C. elegans* gradually increased with the concentration of arecoline (Figure 2A). Quantitative analysis showed that the lipofuscin content of *C. elegans* increased gradually with the concentration of arecoline, and the lipofuscin content of *C. elegans* exposed to 0.2–0.4 mM arecoline was significantly higher than that of the control group by 1.18 and 1.28 times, respectively (Figure 2C).

Oxidative stress not only causes protein damage but also apoptosis. We examined the effect of different concentrations of arecoline exposure on gonad apoptosis induction in *C. elegans* using AO fluorescence staining. Therefore, gonad cell corpses were scored in arecoline-exposed nematodes. As shown in Figure 2A,D, exposure to 0.04 mM of arecoline did not significantly affect the formation of apoptotic cells in the gonads compared with the control; however, 0.2 mM–0.4 mM of arecoline exposure significantly increased the number of cell corpses in *C. elegans*.

Based on the results of the previous study, embryonic cell death was further counted in the N2 and *ced-1*(e1735) strains. Embryos were counted at the comma, 1.5-fold, 2-fold, 3-fold, and 4-fold stages, respectively, where arecoline significantly increased the number of cell corpses in the eggs of N2 and *ced-1*(e1735) in *C. elegans*, and the effect was even more pronounced with continued exposure to the next generation (Figure 2E–H). The significant increase in cell corpses during embryogenesis suggests that arecoline interferes with normal cell survival mechanisms. This could involve the activation of apoptotic pathways or the inhibition of cell survival signals. This result shows that arecoline promotes cell corpses during embryogenesis.

### 2.5. Effect of Arecoline on the Internal Antioxidant Enzyme System of C. elegans

**Figure 3:**
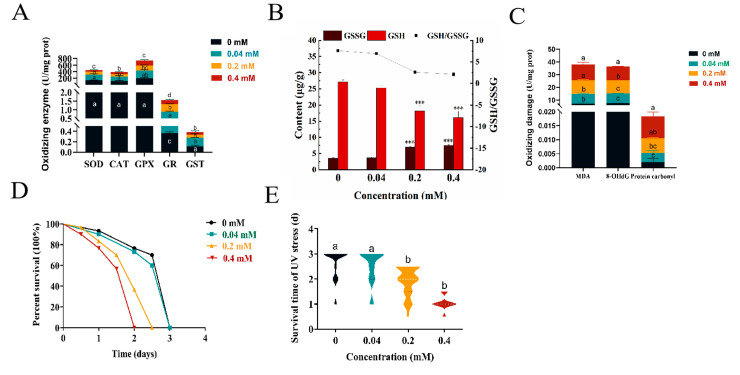
Effect of arecoline on the antioxidant enzyme system and non-enzymatic antioxidant system of *C. elegans*. (**A**) Oxidizing enzyme; (**B**) GSSG and GSH content; (**C**) MDA content, 8-OHdG content and protein carbonyl content; (**D**) UV stress; (**E**) mean survival time of UV stress. Values followed by the different lowercase letters within a row represent the significant difference (Tukey tests, *p <* 0.05). *** Statistical significance at *p* < 0.001. One-way ANOVA with Tukey post hoc test.

To examine the changes in antioxidant enzymes in *C. elegans* after treatment with arecoline, we measured the activities of SOD, CAT, GPX, GR, GST, and GSH/GSSG after treatment. Under 0.2–0.4 mM arecoline treatment, the enzyme activities of SOD, CAT, GPX, GR, GST (Figure 3A) changed; this change indicates that arecoline disrupts the intracellular antioxidant defense mechanisms, potentially leading to increased oxidative stress levels. In addition, as an important detoxification enzyme within cells, the change in glutathione S-transferase (GST) activity following arecoline treatment suggests that the detoxification function of the cells may be affected. GSH/GSSG (Figure 3B) was significantly different from the blank control, except for GPX (Figure 3A), which was significantly lower.

### 2.6. Oxidative Damage by Arecoline on C. elegans

MDA and protein carbonyl content are key indicators for evaluating the degree of oxidative damage, and we found that the MDA and protein carbonyl content increased continuously with increasing treatment concentrations. The MDA and protein carbonyl content in nematodes were significantly higher under the 0.2–0.4 mM treatments compared to the blank control treatment, with the MDA content being 1.48 times and 1.67 times higher than the control (Figure 3C), and the protein carbonyl content being 2.60 times and 3.89 times higher than the control (Figure 3C), respectively. It can be seen that arecoline-induced oxidative damage in *C. elegans* is concentration-dependent and the results are consistent with the trend of ROS changes. 8-OHdG content is a marker for detecting oxidative DNA damage in organisms. We found (Figure 3C) that the 8-OHdG content in *C. elegans* gradually increased with the increase in treatment concentration, with the 8-OHdG content in nematodes under the 0.2–0.4 mM treatments significantly different from the control, at 1.36 and 1.47 times higher than the control, respectively (Figure 3C). Therefore, arecoline promotes intracellular oxidative damage by increasing ROS levels, resulting in significant toxic effects on *C. elegans*.

Since the repair capacity of *C. elegans* was significantly impaired by arecoline, we hypothesize that worms subjected to arecoline treatment may be more susceptible to certain DNA-damaging conditions. To this end, we next investigated the effect of arecoline on nematode resistance to UV damage and the results (Figure 3D,E) showed that the survival ability of nematodes in the 0.2–0.4 mM treatment groups was significantly lower than that of the control group, indicating that the nematode resistance to UV radiation was weakened by arecoline treatment. This result implies that the arecoline-treated worms might be deficient in coping with UV-mediated DNA damage and the protein crosslinking effect.

### 2.7. Effect of Arecoline on the Mitochondria of C. elegans

**Figure 4:**
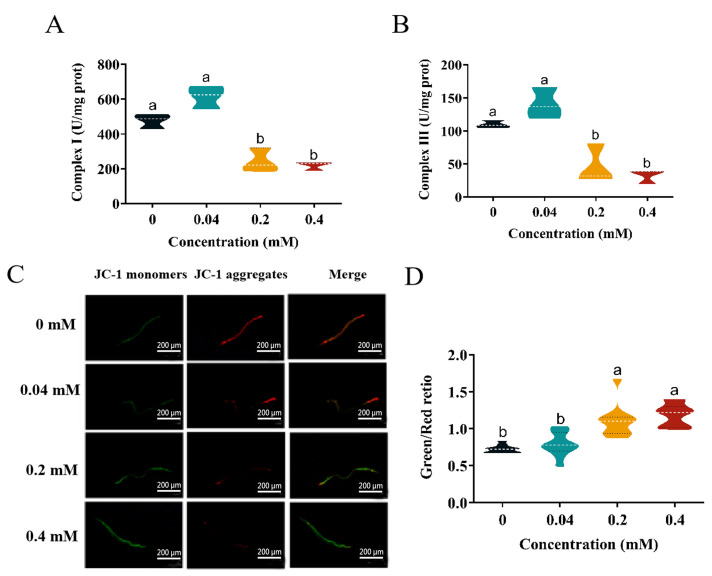
Effect of arecoline on mitochondria in *C. elegans.* (**A**) Electron transport chain complex I; (**B**) electron transport chain complex III. (**C**) MMP. (**D**) Quantitative analysis of green/red fluorescence intensity ratio. Values followed by the different lowercase letters within a row represent the significant difference (Tukey tests, *p <* 0.05). One-way ANOVA with Tukey post hoc test.

In this study, the effects of different concentrations of arecoline exposure on the viability of mitochondrial complexes I and III in *C. elegans* were examined. The results (Figure 4A,B) showed that the enzymatic activities of all complexes in the treated group were elevated at low concentrations compared to the control group, but their enzymatic activities decreased by 54.38% and 70.63% at high concentrations, respectively, and the enzymatic activities of complexes I and III were significantly inhibited, resulting in excessive ROS production.

The effect of different concentrations of arecoline exposure on the mitochondrial membrane potential of *C. elegans* was detected by the JC-1 probe, and the changes in MMP were detected by JC-1 staining. JC-1 exists in two states, monomeric and multimeric, and the emission spectra of both are different. In normal cells, JC-1 aggregates in the mitochondrial matrix and forms polymers that can produce red fluorescence; in early apoptosis, JC-1 cannot aggregate in the mitochondrial matrix when JC-1 is monomeric and can produce green fluorescence. The results (Figure 4C) showed that the green–red fluorescence ratio gradually increased with the increase in arecoline concentration, and the green–red fluorescence ratio was significantly higher than that of the control at the 0.2–0.4 mM arecoline exposure treatments, suggesting that the mitochondrial membrane potential was significantly impaired in *C. elegans*, increasing from 0.73 to 1.18 in vivo (Figure 4D).

### 2.8. Detection of Oxidative Stress Genes by Arecoline in C. elegans

**Figure 5:**
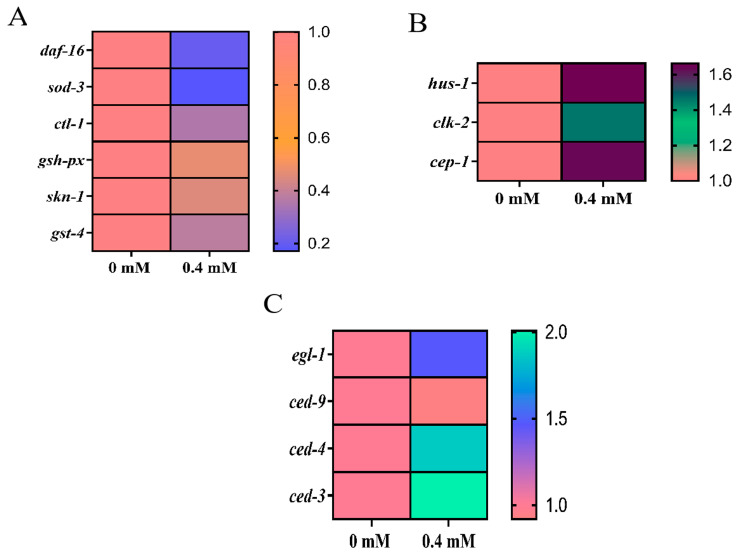
Effect of arecoline on gene expression in *C. elegans*. (**A**) *daf-16* gene, *skn-1* gene and their downstream genes. (**B**) DNA damage genes. (**C**) Apoptosis genes.

The effects of arecoline on the mRNA expression of *daf-16* and its downstream related key genes *sod-3*, *gsh-px*, and *ctl-1* were detected by qRT-PCR. The results (Figure 5A) showed that the mRNA expression of *daf-16* was downregulated by 79.63%; the mRNA expression of *sod-3* was downregulated by 82.75%; and the mRNA expression of *ctl-1* was downregulated by 64.72% compared with the control group. Additionally, the mRNA expression of *gsh-px* was downregulated by 64.72%, and the mRNA expression of *ctl-1* was downregulated by 52.91%. Arecoline-induced downregulation of *daf-16* in *C. elegans* further affected the stress response and longevity pathways. The downregulation of its downstream genes, *sod-3*, *gsh-px*, and *ctl-1*, implies an increased accumulation of superoxide radicals, a decreased capacity to reduce hydrogen peroxide, and an obstruction in the conversion of hydrogen peroxide into water and oxygen. The results (Figure 5A) of the effect of arecoline on the mRNA expression of *skn-1* and its downstream related key gene *gst-4* revealed that the mRNA expression of *skn-1* was significantly downregulated compared with the control group, and the mRNA expression of *gst-4* was also significantly downregulated.

### 2.9. DNA Damage Response by ROS Regulation Involved in the Induction of Apoptosis in C. elegans Cells

The DNA damage response often induces the development of apoptosis. The genes *hus-1* and *clk-2* are key genes for DNA damage-induced apoptosis, and *cep-1*, as a homolog of oncoprotein p53, is a downstream gene of *hus-1* and *clk-2* in nematodes; these genes can respond to the relationship between the DNA damage response pathway and induced apoptosis. Therefore, we further examined the expression of these genes. It was found (Figure 5B) that the relative expression levels of the *hus-1*, *clk-2*, and *cep-1* genes were all significantly increased. It is believed that apoptosis in *C. elegans* is mainly related to the transcriptional regulation of several important genes, namely the *egl-1*, *ced-3,* and *ced-4* apoptosis-promoting genes and an anti-apoptotic gene *ced-9*. The results (Figure 5C) showed that the expression of *egl-1*, *ced-3,* and *ced-4* were significantly upregulated, and the expression of *ced-9* was decreased, but not significantly different from the control.

### 2.10. Results of Pharmacological Mitigation of Arecoline Toxicity to C. elegans

#### 2.10.1. Reduction of Arecoline-Induced ROS Production Levels Using Five Antioxidants

**Figure 6:**
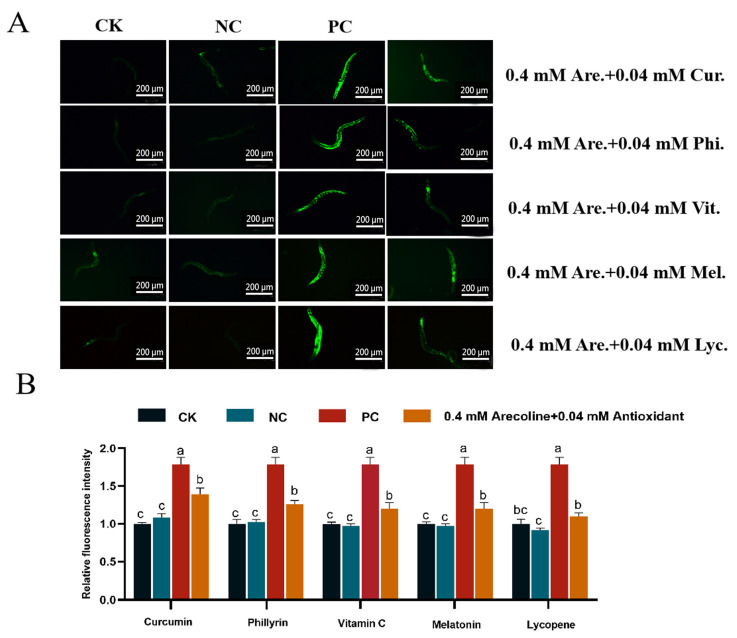
Effect of arecoline + antioxidants on ROS levels in *C. elegans*. (**A**) Fluorescence pictures of ROS. (**B**) Quantification of ROS accumulation. CK: 0 mM; NC: 0.04 mM antioxidants: curcumin; phillyrin; vitamin C; melatonin; lycopene; PC: 0.4 mM arecoline. Values followed by the different lowercase letters within a row represent the significant difference (Tukey tests, *p <* 0.05). One-way ANOVA with Tukey post hoc test.

We observed significant differences in the exposure of L4 stage *C. elegans* to different concentrations of areca nut alkaloids. Therefore, we selected four groups of concentrations, 0 mM, 0.04 mM lycopene (NC), 0.4 mM arecoline (PC), and 0.04 mM lycopene + 0.4 mM arecoline, to expose the L4 stage *C. elegans* and observed the effects on *C. elegans* after 3 days. Based on the results of the preliminary study, we measured the ROS content in *C. elegans* under the treatment of five antioxidants in combination with areca nut alkaloids as the index of concern, in order to screen the best antioxidant for the attenuation experiment. The results (Figure 6A,B) showed that the five antioxidants curcumin, phillyrin, vitamin C, melatonin, and lycopene all reduced the ROS levels by 21.94%, 29.29%, 32.76%, 32.76%, and 38.48%, respectively, compared with the 0.4 mM arecoline treated group, with lycopene having the best effect to reduce intracellular ROS levels. The effect of arecoline + lycopene on *C. elegans* was chosen for the follow-up test.

#### 2.10.2. Longevity Experiment of Arecoline + Lycopene on *C. elegans*

**Figure 7:**
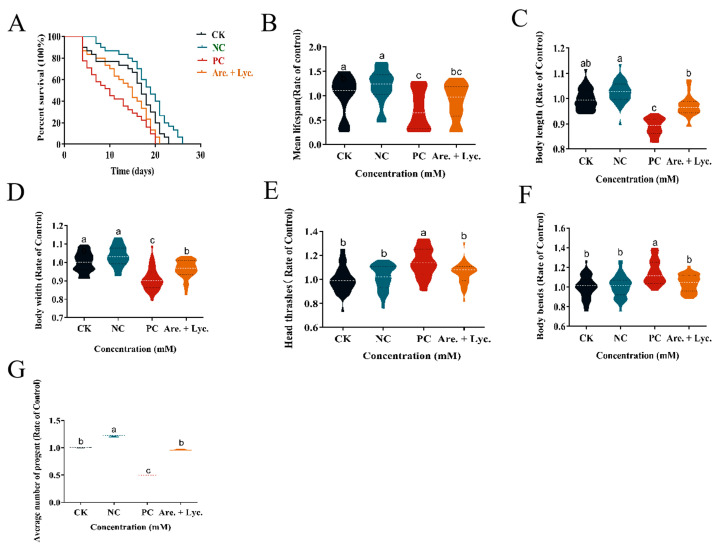
Effect of arecoline + lycopene on the toxicity in *C. elegans*. (**A**) Lifespan. (**B**) Mean lifespan. (**C**) Body length. (**D**) Body width. (**E**) Head thrashes. (**F**) Body bends. (**G**) Average number of progent. CK: 0 mM; NC: 0.04 mM lycopene; PC: 0.4 mM arecoline. Values followed by the different lowercase letters within a row represent the significant difference (Tukey tests, *p <* 0.05). One-way ANOVA with Tukey post hoc test.

The changes in the lifespan of *C. elegans* with different treatments at the standard laboratory conditions of 20 °C were determined, and the results (Figure 7A) showed that the maximum lifespan of the blank control, 0.04 mM lycopene, 0.4 mM arecoline, and 0.4 mM arecoline + 0.04 mM lycopene treatments were 23, 26, 20, and 21 days, respectively, and the average lifespan was 15.33, 18.2, 11, and 13.5 days, respectively (Figure 7B). Compared with the control, the mean lifespan increased by 2.93 days for 0.04 mM lycopene, shortened by 4.33 days for 0.4 mM arecoline, and shortened by 1.83 days for 0.4 mM arecoline + 0.04 mM lycopene. It is evident that lycopene alleviated the effect of arecoline on the lifespan of *C. elegans.*

#### 2.10.3. Effect of Arecoline + Lycopene on Body Length and Body Width of *C. elegans*

The body length and body width of *C. elegans* were measured under different treatments, and the results (Figure 7C,D) showed that the body length and body width of *C. elegans* increased with the addition of 0.04 mM lycopene compared to the group treated with 0.4 mM arecoline, which was not significantly different from the blank control. It can be seen that lycopene alleviated the developmental toxicity of arecoline on the body length and body width of *C. elegans.*

#### 2.10.4. Effect of Arecoline + Lycopene on Head Oscillation and Body Bending of *C. elegans*

The results (Figure 7E,F) showed that the amount of head bobbing and body bending of *C. elegans* was reduced after the addition of 0.04 mM lycopene compared with the group treated with 0.4 mM arecoline, which alleviated the hyperexcitable behavior of *C. elegans* caused by arecoline.

#### 2.10.5. Effect of Arecoline + Lycopene on Egg Production of *C. elegans*

The changes in the egg production of *C. elegans* with different treatments at the standard laboratory condition of 20 °C were determined. The results (Figure 7G) showed that the average egg production of the blank control, 0.04 mM lycopene, 0.4 mM arecoline, and 0.4 mM arecoline + 0.04 mM lycopene were 138.03, 167.67, 68.97, and 133.40 eggs in that order compared to the control group, whereas the mean egg production increased by 29.67 eggs for 0.04 mM lycopene, decreased by 69.07 eggs for 0.4 mM arecoline, and decreased by 4.63 eggs for 0.4 mM arecoline + 0.04 mM lycopene. It can be seen that lycopene alleviated the effect of arecoline soda on the egg-laying of *C. elegans*.

#### 2.10.6. Effect of Arecoline + Lycopene on the Apoptosis Level of *C. elegans*

**Figure 8:**
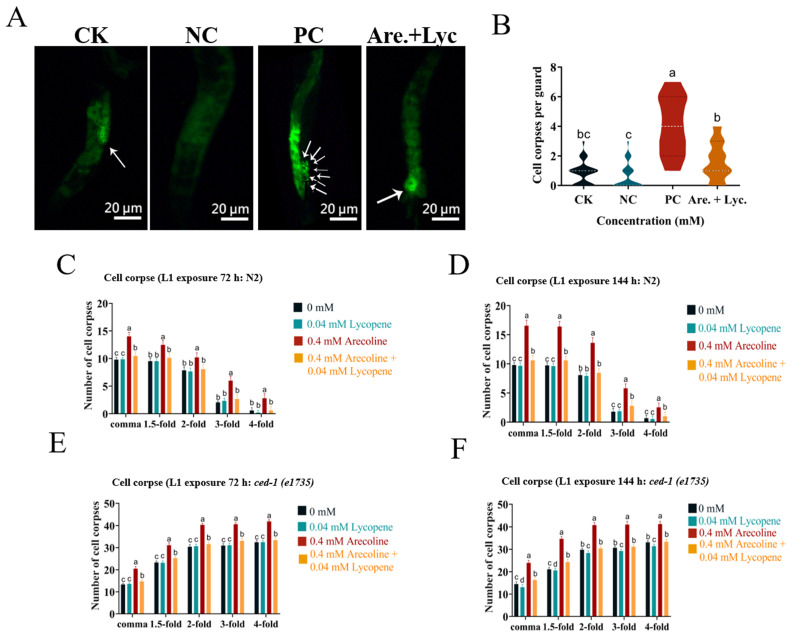
Effect of arecoline + lycopene on apoptosis. (**A**) Gonad apoptosis, the arrows indicate the number of apoptotic cells. (**B**) The gonad apoptosis in the worms was quantified using ImageJ 1.51 software. (**C**,**D**) Cell corpses were counted in N2. (**E**,**F**) Cell corpses were counted in *ced-1*(e1735) strains in *C. elegans.* CK: 0 mM; NC: 0.04 mM lycopene; PC: 0.4 mM arecoline, a < 0.05, b < 0.01, c < 0.001. One-way ANOVA with Tukey post hoc test.

The apoptosis of *C. elegans* gonad cells under different treatments was detected by AO fluorescence staining. The results (Figure 8A,B) showed that cell corpses in *C. elegans* treated with 0.04 mM lycopene and 0.4 mM arecoline + 0.04 mM lycopene were not significantly different from the blank control, indicating that lycopene could alleviate arecoline-induced apoptosis in *C. elegans*.

To investigate whether arecoline treatment affected cell corpses, L1 worms of F0 and F1 generations were treated with 0.04 mM lycopene and 0.4 mM arecoline + 0.04 mM lycopene. The treatment with lycopene significantly alleviated arecoline-induced cell corpses of N2 and *ced-1*(e1735) (Figure 8C–F).

## 3. Discussion

### 3.1. Effect of Arecoline on the Developmental Toxicity of C. elegans

According to a previous study [7], areca nut alkaloids affect the growth and development of zebrafish embryos. In this experiment, *C. elegans* treated with arecoline affected the body length and width of the F0 generation, but did not affect the body length and width of the subsequent generations (Figure 1D,E). Exposure of the parents to penconazole has been reported to cause adverse effects in the parents [9], leading to a reduction in body length and width. However, there was no significant reduction in body length and width in the F1 generation, probably because the presence of food in the absence of drug feeding allowed for the recovery of *C. elegans*, as food could provide the necessary energy for growth, development, and organism repair [17]. In summary, the recovery of the growth and developmental impairment caused by penconazole may be related to food supplementation. In the present study (Figure 1A,B), it was found that the exposure of *C. elegans* to arecoline significantly shortened the mean lifespan in a concentration-dependent manner. In the F1 generation not exposed to the drug solution, the mean lifespan was shortened by 0.2 days, 0.7 days, and 1.2 days, respectively, compared to the control group. Although the pharmaceutical treatment affected the lifespan of the F1 generation, no significant difference was shown.

### 3.2. Effect of Arecoline on the Neurotoxicity of C. elegans

*C. elegans* is the only organism whose entire nervous system has been mapped, allowing every neuron in living animals to be traced [18]. More notably, *C. elegans* has neurotransmitters such as serotonin and acetylcholine, which are associated with humans [19], making it a valuable model for studying the underlying mechanisms of toxicant-induced neurotoxicity. Common evaluation indices for the neurotoxicity of *C. elegans* include motility (frequency of head bobbing and body bending in 20 s) and the content of acetylcholine receptors in the body [20]. Behavior is the first external manifestation of changes in animals to survive and adapt to environmental changes. Based on this, we investigated the effect of arecoline on the head-bobbing and body-bending frequencies of *C. elegans* within 20 s. The results (Figure 1G,H) showed that the head bobbing and body bending of *C. elegans* increased significantly with the increase in arecoline treatment concentration. We examined the effect of arecoline on muscarinic acetylcholine receptors in *C. elegans* and found that muscarinic acetylcholine receptors were activated at 0.2 mM and 0.4 mM concentrations. It has been shown [5] that arecoline induces excitatory responses in rats, and the present study showed that long-term consumption causes *C. elegans* to be in an abnormally hyperactive state, with individual worms showing curling and increased movement, leading to an abnormally high number of head oscillations and body bends in *C. elegans*. By stimulating acetylcholine in the nervous system, arecoline causes an increase in acetylcholine receptor content, prolonging its response rate and resulting in its inability to generate action potentials, thus causing abnormal excitation in nematodes and eventually leading to a toxic reaction. Therefore, we can infer from the above data that the nematode *C. elegans* activates the muscarinic acetylcholine receptors due to the excessive intake of arecoline, which causes a significant increase in the head oscillation frequency and body bending frequency of *C. elegans*. The mechanism of muscarinic acetylcholine receptor activation by arecoline will be the focus of our subsequent study.

### 3.3. Effect of Arecoline on Reproductive Toxicity of C. elegans

Reproductive toxicity is also an important component of the toxicological evaluation. *C. elegans* has 60–80% genetic homology with humans and is a multicellular eukaryote. It is important to carry out studies on the reproductive toxicity of drugs to *C. elegans* because the results of the research can be extended to human reproductive toxicity studies [21]. The number of offspring is considered to be an important indicator in the evaluation of reproductive toxicity [22]. In our study, we found that the number of *C. elegans* offspring gradually decreased with increasing concentrations of arecoline treatment, especially after 0.2–0.4 mM of arecoline exposure, the egg production of *C. elegans* was significantly lower than that of the blank control, and unlike the aforementioned effects on neurotoxicity and developmental toxicity, this effect accumulated to the F1 generation. It can be seen that the excessive intake of arecoline alkaloids affects the reproductive function of *C. elegans* and reduces egg production, and that this effect also extends to the F1 generation.

### 3.4. Induction of Oxidative Stress and Apoptosis by Arecoline in C. elegans

The changes in GSH content in *C. elegans* were measured under different concentrations of arecoline, and it was found (Figure 3B) that the GSH content in nematodes was continuously depleted with increasing treatment concentrations, and that the GSH content in *C. elegans* was significantly lower than that of the blank control under the 0.2 mM and 0.4 mM treatments. The GSSG content increased continuously with higher treatment concentrations, and the ratio of GSH to GSSG decreased gradually, while the ratio of GSH to GSSG was significantly lower than that of the blank control under the 0.2 mM and 0.4 mM treatments. Excessive elevation of ROS levels can cause great harm to the organism [23], and the antioxidant system in the organism plays an important role in scavenging ROS. This antioxidant system is composed of an antioxidant enzyme system and small molecule antioxidants. SOD and CAT are the key enzymes in this antioxidant enzyme system, and SOD can catalyze the generation of H_2_O_2_ from superoxide anions (O^2-^) through a disproportionation reaction, and H_2_O_2_ is then converted to water by CAT [24,25]. Glutathione is credited with the small molecule antioxidant system, and reduced glutathione (GSH) can be catalyzed by glutathione peroxidase (GPx) and react with H_2_O_2_ to generate water [25], thereby reducing the ROS levels in vivo. Glutathione oxide (GSSG) can be reduced to glutathione by glutathione reductase (GR) for GSH regeneration. Changes in the ratio of GSH to GSSG are generally used as a measure of cellular redox status and signaling [26]. We examined the antioxidant enzyme system and glutathione cycle regeneration in *C. elegans* exposed to arecoline, and the increased enzyme activity in the low-concentration treatment group may be related to the stress response of the organism [27]. At high concentrations of the 0.2–0.4 mM agent treatments, the SOD and CAT activities were significantly lower than the control treatment, blocking the antioxidant enzyme system pathway to eliminate ROS. The ratio of GSH to GSSG was significantly lower than that of the blank control, as was GST, and the (GSH:GSSG) ratio is a key indicator to assess the relevant cellular health [28]. The small molecule antioxidant pathway was also significantly affected in the nematodes. The inhibition of mitochondrial complex enzymes I and III in *C. elegans* under the 0.2–0.4 mM treatments resulted in excessive ROS production, and the blockage of the antioxidant pathway led to a significant accumulation of ROS in *C. elegans*. The excessive accumulation of ROS will inevitably cause oxidative damage to the organism. Malondialdehyde (MDA) and protein carbonyl groups reflect the degree of lipid peroxidation and protein oxidation, respectively [29,30,31]. 8-OHdG is a biomarker of oxidative DNA damage [32] while lipofuscin is mainly composed of oxidized lipids and proteins and is an important marker of aging in *C. elegans*. Focusing on these indicators is important to reveal the mechanism of ROS damage. This study (Figure 3C) found that the MDA and protein carbonyl content in *C. elegans* were significantly higher under the 0.2–0.4 mM treatments than the blank control treatment, and the 8-OHdG and lipofuscin content in *C. elegans* were also significantly different from the control. Senescence is the gradual appearance of various physiological, metabolic, and functional changes after the growth and maturation of an organism. Therefore, senescence can also be used to evaluate the toxicity of drugs on organisms at later stages of growth and developmental maturity [33]. It was found that although *C. elegans* did not show acute lethality after being exposed to the pepper toxin, the agent accelerated the aging of *C. elegans*, which was reflected in changes in the nervous system, developmental system, and reproductive system [34]. This is more consistent with a previous study [35]. Meanwhile, the function of mitochondria is highly dependent on MMP, which plays an important role in maintaining biosynthesis and apoptosis as a key parameter of the real-time state of mitochondria [29]. ROS triggers the impairment of MMP and contributes to the continuous production of ROS, which is the main molecule produced in the oxidative stress response of the body and is considered as an important regulator of apoptosis and the after-effects of ROS, as in addition to oxidative damage, ROS also induces apoptosis [8]. The level of apoptosis in *C. elegans* treated with arecoline was measured by AO staining, and it was found that the apoptosis detection signal was significantly enhanced under the 0.2–0.4 mM agent treatments. One study suggested that ROS production might mediate the DNA damage response, and it was found that ROS levels were elevated in *C. elegans* treated with arecoline [36]. To determine the relationship between the induced ROS and DNA damage response checkpoint genes, the 8-OHdG content of agent-treated *C. elegans* was measured, and the results showed (Figure 3C) that the 8-OHdG content increased significantly, indicating some damage to DNA. *daf-16* regulates DNA damage repair, lifespan, and stress response, and it was verified by qRT-PCR that in *C. elegans*, the *daf-16*, *sod-3*, *ctl-1,* and *gsh-px* antioxidant genes were significantly downregulated. *skn-1* mainly regulates lifespan indicators and detoxification, and the *skn-1* gene and its downstream key antioxidant gene, *gst-4*, were found to be significantly downregulated as a result (Figure 5A). This could be one of the reasons for the induction of ROS accumulation, which in turn is a major factor in the induction of apoptosis. The pro-apoptotic genes *egl-1, ced-3, ced-4,* and anti-apoptotic gene *ced-9* are considered key genes for apoptosis in *C. elegans*, and there is a reciprocal interaction between these genes, where *ced-4* exerts apoptosis-inducing effects by activating *ced-3,* and *ced-4* is repressed by *ced-9*, which in turn is repressed by *egl-1* [37]. It was found that the expression of the pro-apoptotic genes *egl-1, ced-3,* and *ced-4* were significantly upregulated, the expression of the anti-apoptotic gene *ced-9* was decreased, and the combined expression of genes resulted in the promotion of apoptosis, validating the experimental data of AO staining.

### 3.5. Toxic Reduction Effect

In order to gain a more comprehensive understanding of the toxicogenic effect of areca nut base and to provide a theoretical basis for the safety of areca nut consumption by consumers, we conducted a small-scale screening to identify antioxidants that can neutralize the arecoline toxicity. The antioxidant selected in this study after screening five antioxidants was lycopene, which further goes to show that the excessive accumulation of ROS is an important factor in the negative changes in the physiological level of *C. elegans*, and that the intake of antioxidants can partially repair the toxic effects caused by arecoline exposure. Lycopene is a carotenoid hydrocarbon pigment [38] that was first found in tomatoes and other red or pink-orange fruits and vegetables such as apricots, cranberries, grapes, guava, papaya, peaches, pink grapefruit, carrots, and watermelon. Lycopene is the most potent in vitro free radical scavenger of all carotenoids, capable of removing ROS and superoxide anions, inhibiting lipid peroxidation, and having a strong antioxidant effect. It has been shown to increase glutathione levels and antioxidant enzyme activity and prevent DNA damage, lipid peroxidation, and other macromolecular compounds through its antioxidant effects [39]. It also has several bioactive functions such as protecting the blood vessels and heart, lowering blood lipids, resisting UV radiation, and improving exercise capacity. In vitro and in vivo studies have shown that lycopene has antioxidant and anti-inflammatory properties [40]. In the context of antioxidant validation, it has been reported in the literature that lycopene goes through the Nrf2 signaling pathway to attenuate oxidative stress [41]. The *skn-1* gene encodes a transcription factor of the mammalian homolog of Nrf2 and activates detoxification responses, whose upregulation promotes resistance to oxidative stress and prolongs the lifespan [42]. Lycopene activates the FOXO signaling pathway for organismal regulation [43], and in addition, Jinde Zhu suggested by KEGG pathway analysis that the FOXO signaling pathway may be related to lycopene [44]. In *C. elegans*, there is a single FOXO transcription factor homolog encoded by the *daf-16* gene. As a central regulator of multiple signaling pathways, DAF-16 integrates these signals and thus regulates a variety of biological processes including longevity, development, stress resistance, and reproduction, and FOXO can be studied using *C. elegans* [45]. With the above reports, it was hypothesized that lycopene could act on the DAF-16/FOXO and SKN-1/Nrf2 pathways in *C. elegans*. Additionally, arecoline with increasing concentration thus inhibits the activity of HUVECs, decreases cell survival, induces morphological and structural changes in cells, and downregulates Nrf2 expression [46]. Therefore, the antioxidant validation of arecoline added to lycopene revealed that arecoline restored the abnormal physiological indicators caused by *C. elegans*. It is hypothesized that areca nut alkaloids may cause oxidative stress by downregulating the *daf-16* and *skn-1*-related gene signaling pathways, leading to ROS accumulation and the induction of apoptosis, which can be subsequently studied in depth.

## 4. Conclusions

This study investigated the effects of arecoline alkaloids on the developmental toxicity, neurotoxicity, reproductive toxicity, oxidative stress, and apoptosis of *C. elegans* at different concentrations. The data showed that 0.04 mM arecoline was less toxic to *C. elegans*, and that the measured indices were not significantly different from the control; 0.2–0.4 mM arecoline was neurotoxic to the F0 generation of *C. elegans*, as reflected by the increase in the number of head oscillations and body bends with increasing concentration. This toxicity was not inherited by the F1 generation; developmental toxicity was reflected in a significant decrease in body length and width and a significant reduction in lifespan, and the effects on body length and width were not inherited by the F1 generation, which was toxic across generations. Reproductive toxicity was reflected in a significant decrease in egg production in the F0 and FI generations, while the activities of mitochondrial complex enzymes I and III were significantly inhibited under the 0.2–0.4 mM exposure treatments. The GSH to GSSG ratio was significantly lower than that of the blank control, the small molecule antioxidant pathway in *C. elegans* was also significantly affected, and the oxidative stress genes were downregulated. In addition, ROS caused the accumulation of ROS in the cells. The excessive intracellular ROS caused oxidative damage in the nematode organism, which was reflected in the significantly higher MDA, protein carbonyl, 8-OHdG, and lipofuscin contents in the nematode compared to the control; at the same time, ROS triggered the impaired mitochondrial membrane potential and induced apoptosis, which led to the significant upregulation of the expression of the pro-apoptotic genes *egl-1*, *ced-3*, and *ced-4*. The toxic effects of ROS could be alleviated by adding lycopene, and the head oscillation frequency and body bending frequency of *C. elegans* treated with 0.4 mM arecoline + 0.04 mM lycopene were significantly lower than those of 0.4 mM arecoline for 20 s. The body length and width were restored, the lifespan was prolonged, the egg production was increased, and the apoptosis level was increased. There was no significant difference between all treatments and the blank control. In conclusion, 0.2–0.4 mM treatments of arecoline were developmentally, neurotoxically, and reproductively toxic to *C. elegans*. The induction of ROS production in *C. elegans* may be the key to its toxic effect. The developmental toxicity (body length, body width, lifespan), neurotoxicity (head bobbing frequency, body bending frequency), and reproductive toxicity (egg production) were all abnormal in the absence of antioxidant treatment compared with the control, but these indices recovered after the addition of antioxidant lycopene and were not significantly different from the control. The target sites of arecoline action are important for follow-up studies.

## 5. Materials and Methods

### 5.1. C. elegans Maintenance

Wild-type *C. elegans* N2, *ced-1*(e1735), and uracil-deficient *Escherichia coli* OP50 were provided by Prof. Rong Di of Rutgers University, Newark, NJ, USA. All strains were grown on nematode growth media (NGM) plates with *E. coli* OP50 at 20 °C.

### 5.2. Arecoline Exposure Assay

Arecoline solutions at concentrations of 0, 0.04, 0.2, and 0.4 mM were prepared using K medium (0.032 M KCl, 0.051 M NaCl, 0.003 M CaCl_2_, 0.003 M MgSO_4_) [47]. Each treatment contained OP50 with an OD600 of 1.5~2 and was stored at 20 °C protected from light. L4 stage *C. elegans* were exposed to different concentrations of arecoline solutions for 72 h and incubated in the dark at 20 °C. Some nematodes were used to measure various toxicity endpoints (motility, lifespan, fecundity, growth, and development), while others were synchronized to the F1 generation and assessed for toxicity without further arecoline exposure. For each concentration, at least 30 nematodes were randomly selected for the experiments.

### 5.3. Antioxidant Detoxification Exposure Assay

To design the detoxification experiment of arecoline, *C. elegans* in the L4 stage were exposed to a mixed solution of 0.4 mM antioxidant and 0.4 mM arecoline. At the same time, a blank control group and an antioxidant-negative control group were set up simultaneously. The culture conditions were the same as those in the arecoline exposure experiment. The drugs included arecoline, curcumin, phillyrin, vitamin C, melatonin, and lycopene, which were purchased from the Shanghai Aladdin Biochemical Technology Limited Company.

### 5.4. Lifespan Assay

Based on previous studies [48], 30 nematodes per group were moved to fresh NGM plates. To prevent egg-laying effects at the end of exposure, these were transferred daily. Nematodes were tapped on the head with a platinum wire, and those not responding were considered dead. Daily survival counts were recorded. The experiment was repeated three times. Statistical analysis and survival curves were created using GraphPad Prism (version 8.0).

### 5.5. Body Length and Body Width

After exposure to the arecoline solution for 72 h, the *C. elegans* samples were washed with M9 buffer (0.043 M NaCl, 0.011 M KH_2_PO_4_, 0.053 M Na_2_HPO_4_-12H_2_O) 3 times and resuspended in 1 mL M9 buffer, then placed in a water bath at 55 °C for 3 min until the body of *C. elegans* was straightened, and then transferred to a slide lined with a 2% agarose gasket after standing at room temperature [34]. The body length and width were measured by imaging using a stereomicroscope.

### 5.6. Head Thrashing and Body Bending

*C. elegans* with 3 days of indicated arecoline treatment were randomly selected and placed on regular NGM plates. A total of 60 μL of the liquid medium was then dropped to submerge *C. elegans*, allowing the *C. elegans* to recover in the liquid medium for 60 s. Then, the number of head oscillations and the number of body bends were measured as previously reported [49]. In brief, head thrashing is defined as the movement of the head in the direction of mid-body bending, while body bending refers to the directional change of the body along the posterior bulb of the pharynx. The number of head thrashes and body bends was recorded within 20 s. After arecoline or antioxidant exposure, at least 30 individuals were examined in each treatment group.

### 5.7. Determination of the Content of Muscarinic Acetylcholine Receptors

After the exposure period, *C. elegans* were collected and homogenized in an extraction buffer to obtain crude protein extracts. The muscarinic acetylcholine receptor content in *C. elegans* was quantified using an ELISA Kit (Jingmei, Jiangsu, China). The total protein concentration of the homogenates was determined using a BCA Kit (Solarbio, Beijing, China) for normalization purposes.

### 5.8. Brood Size Measurement

At the end of the exposure, the nematodes were transferred to fresh NGM plates containing OP50. The plates were replaced with fresh OP50-seeded NGM every 24 h, and the number of eggs laid was counted until egg-laying ceased.

### 5.9. Detection of Arecoline-Induced Oxidative Stress

ROS were detected by adding 1 mL of 10 μM H_2_DCFDA working solution (Solarbio, Beijing, China) to the samples and staining for 120 min at 20 °C protected from light. The supernatant was discarded, washed three times with M9 buffer, and the nematodes were removed and fixed on a 2% agar pad. Approximately 40 μL of 5 mM levamisole hydrochloride was used to paralyze *C. elegans* [34]. Fluorescent images of the samples were examined under an Olympus DM6000B fluorescence microscope (excitation wavelength: 488 nm; emission wavelength: 510 nm), and the fluorescence intensity was quantitated using ImageJ (NIH, Bethesda, MD, USA).

Protein concentrations and mitochondrial and oxidative stress indicators were determined with the BCA (Solarbio, Beijing, China) Protein Concentration Assay Kit and the Solarbio Kit. The mitochondrial stress indicators included complex I and complex III (Solarbio, Beijing, China). Oxidative stress indicators included SOD, CAT, GR, GPX, GSH, and GSSG (Solarbio, Beijing, China) [34].

### 5.10. Detection of Oxidative Damage Index

Oxidative damage indices were assayed and processed similarly to the enzyme activity samples described above. Malondialdehyde and protein carbonyl (Solarbio, Beijing, China) and 8-OHdG (Jingmei, Jiangsu, China) were used to detect the contents of malondialdehyde, protein carbonyl, and 8-OHdG in *C. elegans*; the L4 stage nematodes were randomly transferred to fresh NGM plates after arecoline exposure treatment. The plates were then placed 5 cm below the 254 nm UV lamp and the nematode survival was recorded at 12 h intervals. The survival rate was determined daily until 100% mortality [50].

The lipofuscin content was measured after anesthetizing *C. elegans* with 40 μL of 5 mM levamisole for 20 min. The samples were imaged using a microscope (excitation wavelength: 351 nm; emission wavelength: 420 nm), and the fluorescence intensity was quantitated using ImageJ 1.51 software (NIH, Bethesda, MD, USA) [51].

### 5.11. Measurement of Mitochondrial Membrane Potential

The mitochondrial membrane potential was measured using the potential indicator dye JC-1 [52]. A total of 1 mL of the prepared 10 μM JC-1 solution (Beyotime, Shanghai, China) was added to worms at mixed stages after indicated arecoline treatment. After 2 h of staining in the dark, the *C. elegans* samples were rinsed 2–3 times with M9 to remove the JC-1 solution from the body surface, then transferred to a clean slide with 2% agarose gasket before red fluorescence (emission wavelength of approximately 590 nm) and green fluorescence (emission wavelength of approximately 529 nm) were collected using an Olympus DM6000B fluorescence microscope, and the ratio of red fluorescence to green fluorescence was calculated.

### 5.12. Gonad Apoptosis and Cell Corpse Assay

The worms were suspended in 75 μM acridine orange fluorescent dye at 20 °C for 1 h. Subsequently, the *C. elegans* samples were transferred to NGM plates containing OP50 for 1 h. After 1 h, *C. elegans* was paralyzed with 5 mM levamisole and observed under a fluorescent microscope [53]. The toxicity reduction test was performed as described above. According to the experimental method of David Kokel et al. [54], L1-stage N2 wild-type and *ced-1*(e1735) were exposed to arecoline, and the offspring were exposed continuously for 72 h. The nematodes were picked on NGM medium with fresh OP50 and incubated overnight at 20 °C. Eggs were then picked on agar pads dripped with 1 μL 25 mM NaN_3_ and the number of cell corpses was counted under a DIC microscope (Nikon) for five stages: comma, 1.5-fold, 2-fold, 3-fold, and 4-fold, with *n* = 15 for each stage.

### 5.13. qRT-PCR Analysis

After treatment, 100 mg of *C. elegans* was placed in an ice bath grinder, 1 mL of TRIzol was added rapidly, and the mixture was ground completely. Total RNA was extracted using the TRIzol RNA Kit (Tiangen, Beijing, China), and first-strand cDNA was synthesized using the FastKing cDNA Synthesis Kit (Tolobio, Shanghai, China). qPCR was performed with a qPCR machine (Applied Biosystems) in a 20 μL PCR mix (2×Q3 SYBR qPCR Master Mix Tolobio Kits). The primer sequences for qRT-PCR are shown in Supplementary Table S1. The amplification conditions were as follows: pre-denaturation at 95 °C for 30 s, 95 °C for 10 s, 60 °C for 30 s, and 40 cycles. Melting curves were analyzed as follows: 95 °C for 15 s, 60 °C for 60 s, and 95 °C for 15 s. The actin gene was used as the internal reference gene, and the 2^−ΔΔCt^ method was used to calculate the relative gene expression [55,56].

### 5.14. Statistical Analysis of Data

Plots were made using GraphPad Prism 8 (GraphPad Software). Fluorescence intensity was analyzed using ImageJ 1.51 software. Data variance analysis was performed using GraphPad Prism 5. Data are presented as mean ± SEM. Significance levels are indicated as follows: values followed by different lowercase letters within a row represent the significant difference (Tukey tests, *p <* 0.05). One-way ANOVA with Tukey’s post hoc test was used. To compare the mean differences between the two groups, we used an independent sample *t*-test. For lifespan analysis, the log-rank (Mantel–Cox) test was used to determine statistical significance.

## Data Availability

Dataset available on request from the authors.

## Supplementary Materials

The following supporting information can be downloaded at: https://www.mdpi.com/article/10.3390/toxins16080352/s1, Table S1: Primer sequence of qRT-PCR.
