# Supplementary material for: Oxidative Stress, Oxidative Damage, and Cell Apoptosis: Toxicity Induced by Arecoline in Caenorhabditis elegans and Screening of Mitigating Agents
Source: Toxins (Basel). 2024 Aug 12;16(8):352. doi: 10.3390/toxins16080352 (PMC11359293; doi:10.3390/toxins16080352)
Supplement: Supplementary file 1 [file toxins-16-00352-s001.zip › toxins-3110151-supplementary.pdf]

Table S1 Primer sequence of qRT-PCR

Gene	Primer	Sequence（5’-3’）
act-1	Forward primer	ATGTGTGACGACGAGGTT
	Reverse primer	GAAGCACTTGCGGTGAAC
daf-16	Forward primer	CCAGACGGAAGGCTTAAACT
	Reverse primer	ATTCGCATGAAACGAGAATG
sod-3	Forward primer	CTCCAAGCACACTCTCCCAG
	Reverse primer	TCCCTTTCGAAACAGCCTCG
ctl-1	Forward primer	CGGATACCGTACTCGTGATGAT
	Reverse primer	CCAAACAGCCACCCAAATCA
gsh-px	Forward primer	ATGGCACTTTGGCAGCTCA
	Reverse primer	ACGCGCAAAAAGTAGCAACGC
hus-1	Forward primer	GTTCTGCCCGGCGGACACTG
	Reverse primer	TGTGTCCAATTGACGGCCTGGA
clk-2	Forward primer	GCGAGAGCCACCAAGAGCCG
	Reverse primer	GGCAATGATGCAGGCAGTCCGT
cep-1	Forward primer	TGTATCCAGGCGCAGTT
	Reverse primer	TTGTTTGATGTATGCGTGGAG
egl-1	Forward primer	CACCTTTGCCTCAACCTC
	Reverse primer	TTGGAGCCGATCTCGTAG
ced-3	Forward primer	ACGGGAGATCGTGAAAGC
	Reverse primer	AGAGTTGGCGGATGAAGG
ced-4	Forward primer	CTGATGCTAGCCCGTGTTGT
	Reverse primer	CGTTGCTGGATTTCCACTGC
ced-9	Forward primer	GCTGTTCATCAAAACGCGGA
	Reverse primer	ATCGACCACCGTCTGTTCTG
gst-4	Forward primer	ATGCTCGTGCTCTTGCTGAG
	Reverse primer	GACTGACCGAATTGTTCTCCAT
skn-1	Forward primer	AGTGTCGGCGTTCCAGATTTC
	Reverse primer	GTCGACGAATTGCGAATCA
